# Desempeño del sistema de vigilancia colombiano durante la pandemia de COVID-19: evaluación rápida de los primeros 50 días

**DOI:** 10.7705/biomedica.5582

**Published:** 2020-11-12

**Authors:** Edgar F. Manrique-Hernández, José Moreno-Montoya, Alexandra Hurtado-Ortiz, Franklyn E. Prieto-Alvarado, Álvaro J. Idrovo

**Affiliations:** 1 Departamento de Salud Pública, Escuela de Medicina, Universidad Industrial de Santander, Bucaramanga, Colombia Universidad Industrial de Santander Departamento de Salud Pública Universidad Industrial de Santander Bucaramanga Colombia; 2 Subdirección de Estudios Clínicos, Fundación Santa Fe de Bogotá, Bogotá D.C., Colombia Fundación Santa Fe de Bogotá BogotáD.C Colombia; 3 Dirección de Vigilancia y Análisis del Riesgo en Salud Pública, Instituto Nacional de Salud, Bogotá, D.C., Colombia Dirección de Vigilancia y Análisis del Riesgo en Salud Pública Instituto Nacional de Salud BogotáD.C Colombia

**Keywords:** infecciones por coronavirus, epidemias, notificación de enfermedad, monitoreo epidemiológico, distribuciones estadísticas, Colombia, Coronavirus infections, epidemics, disease notification, epidemiological monitoring, statistical distributions, Colombia

## Abstract

**Introducción.:**

La pandemia de COVID es un desafío para la vigilancia en salud pública y una oportunidad para evaluar sus fortalezas y debilidades en aras de mejorar la respuesta.

**Objetivo.:**

Evaluar el desempeño del sistema de vigilancia en salud pública colombiano durante los primeros 50 días de la pandemia de COVID-19 en el país.

**Materiales y métodos.:**

Se analizaron los datos publicados entre el 6 de marzo y el 24 de abril de 2020 por el Instituto Nacional de Salud y la Organización Mundial de Salud (OMS). Se consideraron en la evaluación: i) la calidad de los datos según la ley de Benford y ii) la oportunidad de la información, medida como la diferencia en fechas entre los datos generados en el Instituto Nacional de Salud y los recogidos en el informe situacional de la OMS. La variabilidad en el cumplimiento de la ley de Benford se evaluó con los valores de p en las pruebas de razón del logaritmo de la verosimilitud, ji al cuadrado o exacta de Moreno.

**Resultados.:**

Hasta el 24 de abril hubo 4.881 casos de COVID-19 en Colombia. En la mayoría de los primeros 50 días se cumplió la ley de Benford, excepto en los primeros días de la epidemia. La diferencia entre los informes del Instituto Nacional de Salud y la OMS ha dependido, en gran medida, de la diferencia en los horarios de cierre de la información.

**Conclusión.:**

En general, el sistema de vigilancia en salud pública colombiano cumplió con la ley de Benford, lo cual sugiere que hubo calidad en los datos. En futuros estudios que comparen el desempeño de los departamentos y distritos se podrá mejorar el diagnóstico de la vigilancia en salud pública del país.

## Abstract (en)

**Introduction:**

The COVID pandemic is a challenge for public health surveillance and an opportunity to assess its strengths and weaknesses to improve the response.

**Objective:**

To evaluate the performance of the Colombian public health surveillance system during the first 50 days of the COVID-19 pandemic in the country.

**Materials and methods:**

We analyzed the data published between March 6 and April 24, 2020, by the *Instituto Nacional de Salud* and the World Health Organization (WHO). We evaluated: i) the quality of the data according to the fulfillment of Benford's law, and ii) the timeliness of the information measured as the difference in dates between the data generated by the *Instituto Nacional de Salud* and WHO's situational reports. We assessed the fulfillment of Benford's law using the p values of the log-likelihood ratio, the chi square or Moreno's exact tests.

**Results:**

Until April 24 there were 4,881 cases of COVID-19 in Colombia. During most of the first 50 days of the pandemic, Benford's law was fulfilled except the first days of the epidemic. The difference between *Instituto Nacional de Salud* and WHO reports largely depends on the different reporting times.

**Conclusion:**

In general, the Colombian public health surveillance system fulfilled Benford's law suggesting that there was quality in the data. Future studies comparing the performance of the departments and districts will improve the diagnosis of the Colombian surveillance system.

La vigilancia en salud pública es una herramienta útil para la monitorización de enfermedades, particularmente las infecciosas, y se originó en el puerto marítimo de Venecia, Italia, en 1348, cuando tres guardianes de salud pública tenían como función detectar e impedir el desembarco de personas con peste negra. Este tipo de iniciativas dieron origen a estrategias preventivas como la cuarentena, la detección temprana de casos y el aislamiento ^1^. Con el paso de los años, dicha vigilancia se ha ido retinando hasta convertirse en un elemento esencial para el manejo sanitario y la seguridad en cualquier país, especialmente en cuanto a las enfermedades infecciosas con potencial pandémico ^2^^,^^3^.

Según los *Centers for Disease Control and Prevention* (CDC) de los Estados Unidos, la vigilancia en salud pública consiste en la "recolección continua y sistemática, el análisis, la interpretación y la difusión de datos sobre un evento relacionado con la salud para su uso en acciones de salud pública encaminadas a reducir la morbilidad y la mortalidad y mejorar salud" ^4^. Si bien existen otras definiciones, todas con elementos comunes, la vigilancia en salud pública es la herramienta fundamental para el control de las epidemias, especialmente en lo tocante a los tratamientos o las vacunas para su prevención ^5^^,^^6^, como en el caso de la pandemia de COVID-19.

En Colombia, la vigilancia en salud pública no ha sido ajena a los desarrollos técnico-científicos y ha seguido fundamentalmente la estrategia de formación de epidemiólogos de campo del CDC ^7^. Un hito en la historia de la epidemiología de campo en Colombia ocurrió en 1993, cuando se inició la consolidación del grupo del Instituto Nacional de Salud encargado del manejo y el estudio de epidemias bajo parámetros modernos.

A partir de entonces, han sido innumerables los casos de manejo de conglomerados y epidemias que pueden considerarse como éxitos de la epidemiología de campo colombiana ^8^. Es el caso del manejo de pandemias como la de la influenza A(H_1_N_1_) en el 2009, que permitió hacer una evaluación de la percepción de las competencias de los epidemiólogos y profesionales de salud pública para manejar este tipo de emergencias sanitarias ^9^ y contrastarla con los análisis objetivos del desempeño de la vigilancia en salud pública de otros países ^10^. En ese momento pudieron detectarse, asimismo, algunas deficiencias que han tenido diversos cursos de solución con el paso del tiempo.

En ese sentido, el presente trabajo tuvo como objetivo evaluar el desempeño del sistema de vigilancia en salud pública colombiano durante los primeros 50 días de la pandemia de COVID-19 en el país.

## Materiales y métodos

Se analizaron los datos oficiales del sistema colombiano disponibles en la página web del Instituto Nacional de Salud (https://www.ins.gov.co/Noticias/Paginas/Coronavirus.aspx) y los informes situacionales de la Organización Mundial de la Salud (OMS) (https://www.who.int/emergencies/diseases/novel-coronavirus-2019/situation-reports/). Si bien el sistema de vigilancia en salud pública está activo todo el tiempo, se incluyeron en este análisis los datos a partir del día en que fue diagnosticado el primer caso de COVID-19, el cual se reportó en Bogotá el 6 de marzo de 2020.

Se entiende que la evaluación cobija todos los procesos involucrados en las labores de los epidemiólogos de campo en los equipos de respuesta inmediata, así como el procesamiento de muestras en el laboratorio y la generación de los reportes situacionales que se divulgan a nivel nacional e internacional.

Se consideraron dos indicadores: i) la calidad de los datos según el cumplimiento de la ley de Benford, y ii) la oportunidad de la información. Este último indicador se consideró en atención a las necesidades internacionales o fronterizas de vigilancia de los fenómenos migratorios en el curso de una pandemia.

### La ley de Benford

Esta ley matemática, también denominada "ley de los primeros dígitos" "ley de Newcomb-Benford" o "ley de los números anómalos" ^11^, señala que en un conjunto de números, aquellos que empiezan con el número 1 son más frecuentes que los que empiezan con otros dígitos, siguiendo un orden decreciente que se puede expresar como P(d)=log [1+(1=d)] d=1, 2, . . . , 9, donde una serie de números P(d) corresponde a la probabilidad de que un dígito sea el número inicial ^12^^,^^13^.

Este método matemático ha sido particularmente útil y exitoso en la detección de datos fraudulentos ^14^^,^^15^. En el campo de la vigilancia en salud pública, se usó por primera vez durante la pandemia de influenza A(H_1_N_1_) y, después, en las epidemias de dengue en Paraguay ^16^, de Zika en América ^17^ y de COVID-19 en China ^18^.

La oportunidad de la información se evaluó calculando la diferencia entre el tiempo en que se informaba el número de casos y fallecidos de Colombia en el portal del Instituto Nacional de Salud y el tiempo en que aparecía dicha información en el portal de la OMS. Dada la importancia de contar con información integral y oportuna a nivel mundial para definir las acciones multinacionales, este indicador puede entenderse como una evaluación de la magnitud del "rezago temporal".

### Métodos estadísticos

Para evaluar el nivel de cumplimiento de la distribución descrita por la ley de Benford en los datos observados (casos diarios acumulados), se usaron las pruebas de razón del logaritmo de la verosimilitud y de ji al cuadrado mediante la macro *digdis* desarrollada por Ben Jann (ETH, Zurich). Para verificar el cumplimiento de la ley de Benford en los datos de las muestras pequeñas durante los primeros días de reporte, se usó la prueba exacta de Moreno ^19^, ya que resultó tener un mejor desempeño que la prueba de Kuiper ^20^. Dichos análisis se hicieron con el programa estadístico Stata 14™ (Stata Corporation, USA) o el programa R.

## Resultados

En el cuadro 1 se encuentran los resultados del análisis del cumplimiento de la ley de los primeros dígitos en el número de casos acumulados día a día desde el 6 de marzo hasta el 24 de abril de 2020.

**Cuadro 1:** Cumplimiento de la ley de Benford de cada uno de los reportes de número de casos de COVID-19 en Colombia (casos acumulados)

Reporte (n)	Fecha	Casos acumulados	Primeros dígitos									Exacta de Moreno	Razón del logaritmo de la verosimilitud	χ^2^
			1	2	3	4	5	6	7	8	9			
1	06/03/2020	1	1	0	0	0	0	0	0	0	0	0,3010		
2	07/03/2020	1	2	0	0	0	0	0	0	0	0	0,0906		
3	08/03/2020	1	3	0	0	0	0	0	0	0	0	0,0272		
4	09/03/2020	3	3	0	1	0	0	0	0	0	0	0,0136		
5	10/03/2020	3	3	0	2	0	0	0	0	0	0	0,0042		
6	11/03/2020	9	3	0	2	0	0	0	0	0	1	0,0011		
7	12/03/2020	9	3	0	2	0	0	0	0	0	2	0,0001		
8	13/03/2020	16	4	0	2	0	0	0	0	0	2	0,0001	0,0920	0,0936
9	14/03/2020	24	4		2	0	0	0	0	0	2	0,0001	0,2121	0,2129
10	15/03/2020	45	4		2		0	0	0	0	2	0,0001	0,3434	0,3544
11	16/03/2020	57	4		2			0	0	0	2	0,0001	0,4986	0,5055
12	17/03/2020	75	4		2			0		0	2		0,6284	0,6243
13	18/03/2020	102	5		2			0		0	2		0,5890	0,6356
14	19/03/2020	128	6		2			0		0	2		0,5232	0,6081
15	20/03/2020	158	7		2			0		0	2		0,4413	0,5505
16	21/03/2020	210	7	2	2			0		0	2		0,5710	0,6953
17	22/03/2020	235	7	3	2			0		0	2		0,6058	0,7607
18	23/03/2020	306	7	3	3			0		0	2		0,5775	0,7604
19	24/03/2020	378	7	3	4			0		0	2		0,4891	0,6793
20	25/03/2020	470	7	3	4	2		0		0	2		0,5422	0,7548
21	26/03/2020	491	7	3	4	3		0		0	2		0,4947	0,7261
22	27/03/2020	539	7	3	4	3	2	0		0	2		0,5255	0,7768
23	28/03/2020	608	7	3	4	3	2			0	2		0,8306	0,9140
24	29/03/2020	702	7	3	4	3	2		2	0	2		0,8156	0,9097
25	30/03/2020	798	7	3	4	3	2		3	0	2		0,6969	0,7875
26	31/03/2020	906	7	3	4	3	2		3	0	3		0,5296	0,5611
27	01/04/2020	1.065	8	3	4	3	2		3	0	3		0,5430	0,6018
28	02/04/2020	1.161	9	3	4	3	2		3	0	3		0,5374	0,6214
29	03/04/2020	1.267	10	3	4	3	2		3	0	3		0,5157	0,6219
30	04/04/2020	1.406	11	3	4	3	2		3	0	3		0,4807	0,6050
31	05/04/2020	1.485	12	3	4	3	2		3	0	3		0,4354	0,5727
32	06/04/2020	1.579	13	3	4	3	2		3	0	3		0,3833	0,5273
33	07/04/2020	1.780	14	3	4	3	2		3	0	3		0,3277	0,4714
34	08/04/2020	2.054	14	4	4	3	2		3	0	3		0,4214	0,5809
35	09/04/2020	2.223	14	5	4	3	2		3	0	3		0,4816	0,6589
36	10/04/2020	2.473	14	6	4	3	2		3	0	3		0,5087	0,7036
37	11/04/2020	2.709	14	7	4	3	2		3	0	3		0,5065	0,7176
38	12/04/2020	2.776	14	8	4	3	2		3	0	3		0,4801	0,7036
39	13/04/2020	2.852	14	9	4	3	2		3	0	3		0,4348	0,6634
40	14/04/2020	2.979	14	10	4	3	2		3	0	3		0,3763	0,5990
41	15/04/2020	3.105	14	10	5	3	2		3	0	3		0,3981	0,6410
42	16/04/2020	3.233	14	10	6	3	2		3	0	3		0,3875	0,6437
43	17/04/2020	3.439	14	10	7	3	2		3	0	3		0,3518	0,6098
44	18/04/2020	3.621	14	10	8	3	2		3	0	3		0,2995	0,5429
45	19/04/2020	3.792	14	10	9	3	2		3	0	3		0,2394	0,4499
46	20/04/2020	3.977	14	10	10	3	2		3	0	3		0,1798	0,3431
47	21/04/2020	4.149	14	10	10	4	2		3	0	3		0,2078	0,3993
48	22/04/2020	4.356	14	10	10	5	2		3	0	3		0,2107	0,4200
49	23/04/2020	4.561	14	10	10	6	2		3	0	3		0,1931	0,4038
50	24/04/2020	4.881	14	10	10	7	2		3	0	3		0,1622	0,3554

Dado que las pruebas estadísticas para evaluar el cumplimiento de la ley de Benford son pruebas de bondad de ajuste, los valores de p más pequeños sugieren que esta no se cumplió. Debido a que se contaba con pocas observaciones en los primeros días, para estos datos se usó exclusivamente la prueba exacta de Moreno, cuyos resultados sugieren un buen desempeño el primer día; después disminuyó la calidad de los datos hasta que entre los días 3 y 11 de la vigilancia (8 a 16 de marzo) no se cumplió la ley de Benford.

**Figura 1:**
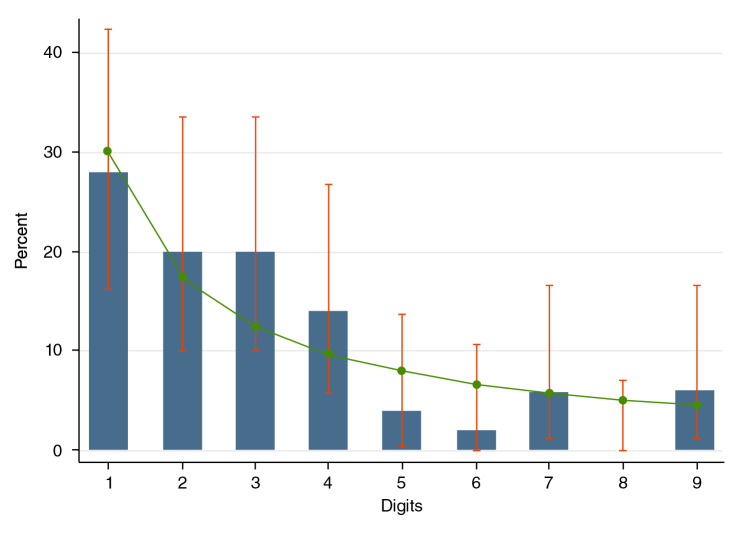
Frecuencia de los primeros dígitos en la distribución de la ley de Benford reportados en el último día analizado

Para los datos a partir del día 8 de la vigilancia (13 de marzo) la evaluación se hizo con las pruebas de razón del logaritmo de la verosimilitud y de ji al cuadrado, las cuales registraron una mejoría en el cumplimiento de la ley de Benford; los días de mejor desempeño fueron el 28 y el 29 de marzo (p>0,80). Con valores más bajos, aunque estadísticamente satisficieron la ley de Benford, aparecen los días 20, 23 y 24 de abril, con valores de p entre 0,15 y 0,20. En el cuadro 2 y la figura 1 se presentan en detalle los resultados del último día de vigilancia incluido en este análisis.

**Cuadro 2:** Cumplimiento de la ley de Benford en el día 50 de epidemia de COVID-19 en Colombia (casos acumulados)

Primer dígito	Conteo	%	P
1	14	28	0,8777
2	10	20	0,7095
3	10	20	0,1295
4	7	14	0,3322
5	2	4	0,4338
6	1	2	0,2592
7	3	6	0,7659
8	0	0	0,1838
9	3	6	0,4987

**Figura 2:**
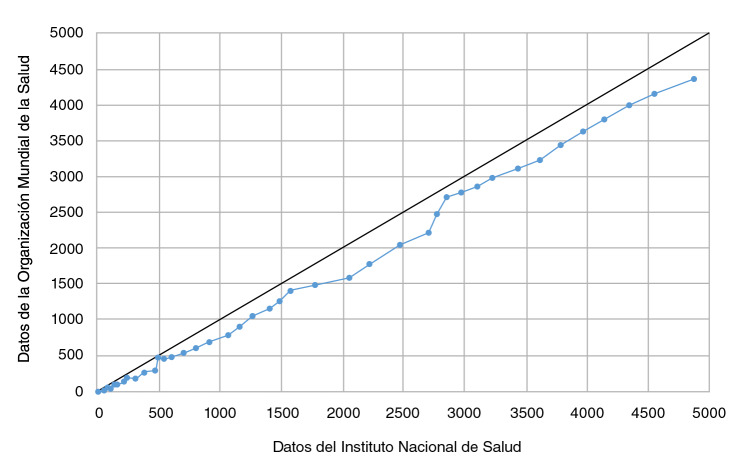
Diferencia en los casos acumulados diarios de COVID-19 reportados en los portales del Instituto Nacional de Salud y la Organización Mundial de la Salud (6 de marzo y el 24 de abril de 2020).

En la figura 2 se observan los casos acumulados según lo reportado por el Instituto Nacional de Salud y la OMS para Colombia en las mismas fechas. Como se puede apreciar, la línea azul se encuentra siempre por debajo de la línea negra, lo que equivale a una igualdad en los reportes, por lo que se concluye que hay un claro rezago (de dos días usualmente) en la información publicada por la OMS.

## Discusión

Los hallazgos de estos análisis sugieren que los primeros días de la vigilancia del COVID-19 en territorio colombiano fueron los más difíciles y la calidad de los datos no fue óptima. En los dos primeros días se cumplió la ley de Benford, al no registrarse más que un solo caso; luego, entre el día 3 y el día 11 (8 a 16 de marzo), la ley de los primeros dígitos no se cumplió porque durante tres días hubo un único caso y se pasó directo a tres y de allí a nueve casos de COVID-19. Este es un comportamiento esperable dado que apenas se empezaban a afinar las actividades de la vigilancia en salud pública.

La problemática de los primeros días de epidemia en Colombia se relacionó con las dificultades para garantizar la continuidad de la oferta de la prueba por no disponerse de suficientes reactivos, de equipos para la operación en serie, de infraestructura a nivel regional y local para descentralizar el diagnóstico, y por el proceso particular de la prueba estandarizada por la OMS, entre otros.

En los primeros días también se detectó que se priorizaba la prueba sobre el registro de la vigilancia, situación que se fue ajustando rápidamente. Además, algunos niveles locales asumieron la gobernanza para organizar más estructuradamente en su territorio la notificación, la investigación epidemiológica de campo, el estudio de contactos y la recolección de muestras. Después de esos primeros días, en general, los datos de la vigilancia en salud pública sugieren que se ha cumplido con la ley de Benford, aunque con fluctuaciones. El cumplimiento de la ley de Benford a juzgar por los valores de p de la prueba de razón del logaritmo de la verosimilitud muestra altibajos.

Al poner estos datos en el contexto que brindan los análisis del desempeño de la vigilancia en salud pública durante las epidemias de influenza A(H_1_N_1_), de Zika y ahora de COVID-19 (cuadro 3), se observa que su ejercicio en Colombia se ha consolidado y que el componente basado en el laboratorio ^21^ ha tenido mejor desempeño cuando los agentes infecciosos son transmitidos de persona a persona por vía respiratoria que cuando estos son transmitidos por insectos vectores. Sin embargo, cabe destacar que el mejor desempeño del sistema se registró con los casos probables de Zika valorados a partir de criterios clínicos y epidemiológicos, y relacionado con un mayor reconocimiento de cuadros sindrómicos específicos, como el de Guillain-Barré, y el de los defectos congénitos relacionados ^22^.

**Cuadro 3:** Cumplimiento de la ley de Benford (valores de p en la prueba de razón del logaritmo de la verosimilitud) de la vigilancia en salud pública en Colombia durante las epidemias de influenza A(H1N1), Zika y COVID-19

Casos	Influenza A(H1N1) 2009	Virus Zika 2016	Virus COVID-19 2020*
Confirmados (semanas)	0,190 (25)	0,068 (42)	0,162 (50)
Sospechosos (semanas)	ND	0,467 (41)	ND

* Incluye solamente los primeros 50 días a partir del diagnóstico del primer caso en Colombia.

En cuanto a la oportunidad de la información, se pudo observar un rezago temporal importante entre el Instituto Nacional de Salud y la OMS, que corresponde, en gran medida, a las diferentes horas de cierre de cada informe situacional. La OMS suele hacer su cierre de datos hacia las 03:00 hora de Colombia (10:00, hora de verano de Europa Central), en tanto que el Instituto Nacional de Salud ha variado las horas, siendo más frecuente en las tardes (14:00 a 18:00) de cada día.

En este sentido, es interesante anotar que el *Center for Systems Science and Engineering* (CSSE) de la *Johns Hopkins University* organizó un tablero interactivo en internet (https://coronavirus.jhu.edu/) que disminuye notoriamente este rezago temporal, pues recibe directamente de cada país o territorio los datos y va actualizando la información, lo que permite tenerla casi en tiempo real ^23^. Este tablero ha sido la fuente más confiable en tiempo real de la pandemia alrededor del mundo.

Uno de los avances más importantes del sistema de vigilancia colombiano es la disponibilidad de datos abiertos (https://www.ins.gov.co/Noticias/Paginas/Coronavirus.aspx), que permiten verificar la oportunidad del sistema en términos del reporte de los casos confirmados, casos que han sido investigados desde la condición probable en términos de la contención.

Los hallazgos aquí descritos no corresponden a una evaluación completa de un sistema de vigilancia en salud pública. Otros elementos como la simplicidad, la flexibilidad, la aceptabilidad, la sensibilidad, la representatividad, la estabilidad y el valor predictivo positivo ^24^ deben contemplarse para tener una visión integral. Sin embargo, este análisis preliminar de la calidad de los datos y de su oportunidad en etapas iniciales de una epidemia resulta muy útil, pues permite realimentar rápidamente el sistema de vigilancia, con el fin de adoptar medidas correctivas si es el caso.

Dado que en Colombia son muy diversas las capacidades en salud pública y epidemiología, en futuros análisis se podrán evaluar los sistemas de vigilancia en los departamentos y distritos para así determinar acciones a ese nivel que favorezcan el mejoramiento continuo de la vigilancia en salud pública.
